# Evaluating single-subject study methods for personal transcriptomic interpretations to advance precision medicine

**DOI:** 10.1186/s12920-019-0513-8

**Published:** 2019-07-11

**Authors:** Samir Rachid Zaim, Colleen Kenost, Joanne Berghout, Francesca Vitali, Helen Hao Zhang, Yves A. Lussier

**Affiliations:** 1 The Center for Biomedical Informatics & Biostatistics of the University of Arizona Health Sciences, 1230 N. Cherry Ave, Tucson, AZ 85721 USA; 2 The Department of Medicine, College of Medicine Tucson, 1501 N. Campbell Ave, Tucson, AZ 85724-5035 USA; 3 The Graduate Interdisciplinary Program in Statistics, The University of Arizona, 617 N. Santa Rita Ave, Tucson, AZ 85721 USA; 4 The Center for Applied Genetic and Genomic Medicine, 1295 N. Martin, Tucson, AZ 85721 USA; 5 The Department of Mathematics, College of Sciences, The University of Arizona, 617 N. Santa Rita Ave, Tucson, AZ 85721 USA; 6 The University of Arizona Cancer Center, 3838 N. Campbell Ave, Tucson, AZ 85719-1454 USA

**Keywords:** Single-subject studies, Precision medicine, Genomic medicine, Medical genomics, N-of-1, Transcriptome, N-of-1 studies

## Abstract

**Background:**

Gene expression profiling has benefited medicine by providing clinically relevant insights at the molecular candidate and systems levels. However, to adopt a more ‘precision’ approach that integrates individual variability including ‘omics data into risk assessments, diagnoses, and therapeutic decision making, whole transcriptome expression needs to be interpreted meaningfully for single subjects. We propose an “all-against-one” framework that uses biological replicates in isogenic conditions for testing differentially expressed genes (DEGs) in a single subject (ss) in the absence of an appropriate external reference standard or replicates. To evaluate our proposed “all-against-one” framework, we construct reference standards (RSs) with five conventional *replicate-anchored analyses* (NOISeq, DEGseq, edgeR, DESeq, DESeq2) and the remainder were treated separately as single-subject sample pairs for *ss analyses* (without replicates).

**Results:**

Eight *ss methods* (NOISeq, DEGseq, edgeR, mixture model, DESeq, DESeq2, iDEG, and ensemble) for identifying genes with differential expression were compared in Yeast (parental line versus snf2 deletion mutant; *n* = 42/condition) and a MCF7 breast-cancer cell line (baseline versus stimulated with estradiol; *n* = 7/condition). Receiver-operator characteristic (ROC) and precision-recall plots were determined for eight ss methods against each of the five RSs in both datasets. Consistent with prior analyses of these data, ~ 50% and ~ 15% DEGs were obtained in Yeast and MCF7 datasets respectively, regardless of the RSs method. NOISeq, edgeR, and DESeq were the most concordant for creating a RS. Single-subject versions of NOISeq, DEGseq, and an ensemble learner achieved the best median ROC-area-under-the-curve to compare two transcriptomes without replicates regardless of the RS method and dataset (> 90% in Yeast, > 0.75 in MCF7). Further, distinct specific single-subject methods perform better according to different proportions of DEGs.

**Conclusions:**

The “all-against-one” framework provides a honest evaluation framework for single-subject DEG studies since these methods are evaluated, by design, against reference standards produced by unrelated DEG methods. The ss-ensemble method was the only one to reliably produce higher accuracies in all conditions tested in this conservative evaluation framework. However, single-subject methods for identifying DEGs from paired samples need improvement, as no method performed with precision> 90% and obtained moderate levels of recall.

http://www.lussiergroup.org/publications/EnsembleBiomarker

## Background

Gene expression profiling has benefited medicine by characterizing cellular states throughout development and differentiation, describing the pathological processes occurring during disease and providing clinically relevant insights at the molecular candidate and systems levels. As medicine moves to adopt a more ‘precision’ approach that integrates individual variability including ‘omics data into risk assessments, diagnoses, and therapeutic decision making, whole transcriptome expression analyses using technologies such as RNA-Seq are poised to become foundational methods [1]. Still, there are issues to resolve before this promise can be realized; most related to data analysis and interpretation rather than data collection, though all areas can still be better optimized. Major areas for computational analytical methods improvements include (i) the development of a well-validated reference standard, thoroughly vetted and solidly benchmarked for a given investigation, and (ii) the ability to confidently make individual-level inferences from transcriptomic data.

To the last point, the majority of *differentially expressed gene* (DEG) analysis methods currently available have been designed to make inferences at the population level about diseases or conditions, not for individual patients. These experiments and analytical approaches seek to define and characterize the common and consensus processes that differentiate or underlie two (or more) states. In basic research using model organisms, establishing controls over genotype and experimental parameters allows genotype-level inference by using a two-group comparison with three or more replicates per group [2]. In clinical research using human subjects, however, the genotypic and lived experience diversity of each subject introduces substantial biological variability and noise into expression data. This then requires tens to thousands of genotype-distinct replicate samples to draw inferences about the population(s) and condition(s) of interest, but simultaneously ignores or prohibits individual-level variation and inferences unless they can be classified according to stratification patterns common enough to be noticed [3]. To adapt the tools designed for populations into tools appropriate for individual-level inference requires either the use of replicates (mimicking the style of a model organism experiment and reducing the cross-sample noise to primarily stochastic and technical factors), a priori distribution and parameter assumptions, or data-derived models to create an expected distribution useful for comparison. However, in practice, it is not cost-effective and often entirely infeasible to obtain replicate samples from clinical procedures. Since DEG analysis methods were validated using replicates [3, 4], there remains a need to learn how well a DEG method designed for identifying differential expression would perform in real-world conditions and when replicates are unavailable (ss-DEG Methods).

Novel methodological advances designed with single subjects in mind have begun to be proposed [3, 4]. While accurately discovering DEGs between two RNA-Seq samples remains a challenge and insufficiently studied [3, 4], methods identifying *differentially expressed gene sets and pathways* between two transcriptomes applicable to single-subject studies have been reproducibly demonstrated as feasible [3, 4] in simulations [5], retrospective studies in distinct datasets [5–10], cellular assays [11, 12], as well as in one clinical classifier [13] ( Table 1). These comprehensive validations of gene set/pathway-level methods established the feasibility of single-subject interpretation of the transcriptomes and stimulate further investigations to improve more precise methods for determining the underlying differentially expressed genes. However, transcriptional dynamics operating and validated at the gene set or pathway-level cannot straightforwardly be deconvoluted to identify specific transcripts altered in a single subject. A recent study provides a comparison of accuracy for five ss-DEGs methods using computer simulations of several data models with genomic dysregulation ranging from 5 to 40% of DEGs [14]. A partial independent biological validation was conducted for one ss-DEG method, NOIseq [15], confirming 400 DEG signals by qPCR. Yet, and to the best of our knowledge, no study has comprehensively validated nor compared the accuracies of ss-DEG methods using biological or clinical datasets on a transcriptome scale. In addition, no framework has been proposed on how to conduct such a comprehensive validation.

We and others [16] propose that there is a knowledge gap in the field with regards to optimizing the operating characteristics of the state-of-the-art RNA-Seq analytics for precision medicine: what are the best ss-DEG methods for interrogating two RNA-Seq samples from one patient taken in two different conditions without replicates? Reliable and accurate of ss-DEG methods can have practical utility. For example, the comparison of affected versus unaffected samples (e.g., cancer versus non-cancer) can provide valuable insight into the genetic variables involved in a disease’s pathophysiology and therapeutics. Similarly, using a patient’s healthy tissue as its baseline to compare treated tissue or evolution over time provides another framework to design analytics and assays for precision medicine.

We thus designed this study under the following premise: *isogenic (genome matched) biological replicates can provide a framework for testing single-subject methods in the absence of an externally valid reference standard.* In this study, we aim to identify the best-performing techniques and parameters in absence of replicates of distinct single-subject (ss) methods predicting differentially expressed genes (DEGs). In addition, we hypothesized, implemented, and evaluated an ensemble method as possibly more robust across different conditions of application for determining DEGs in single subjects.

## Methods

**Fig. 1:**
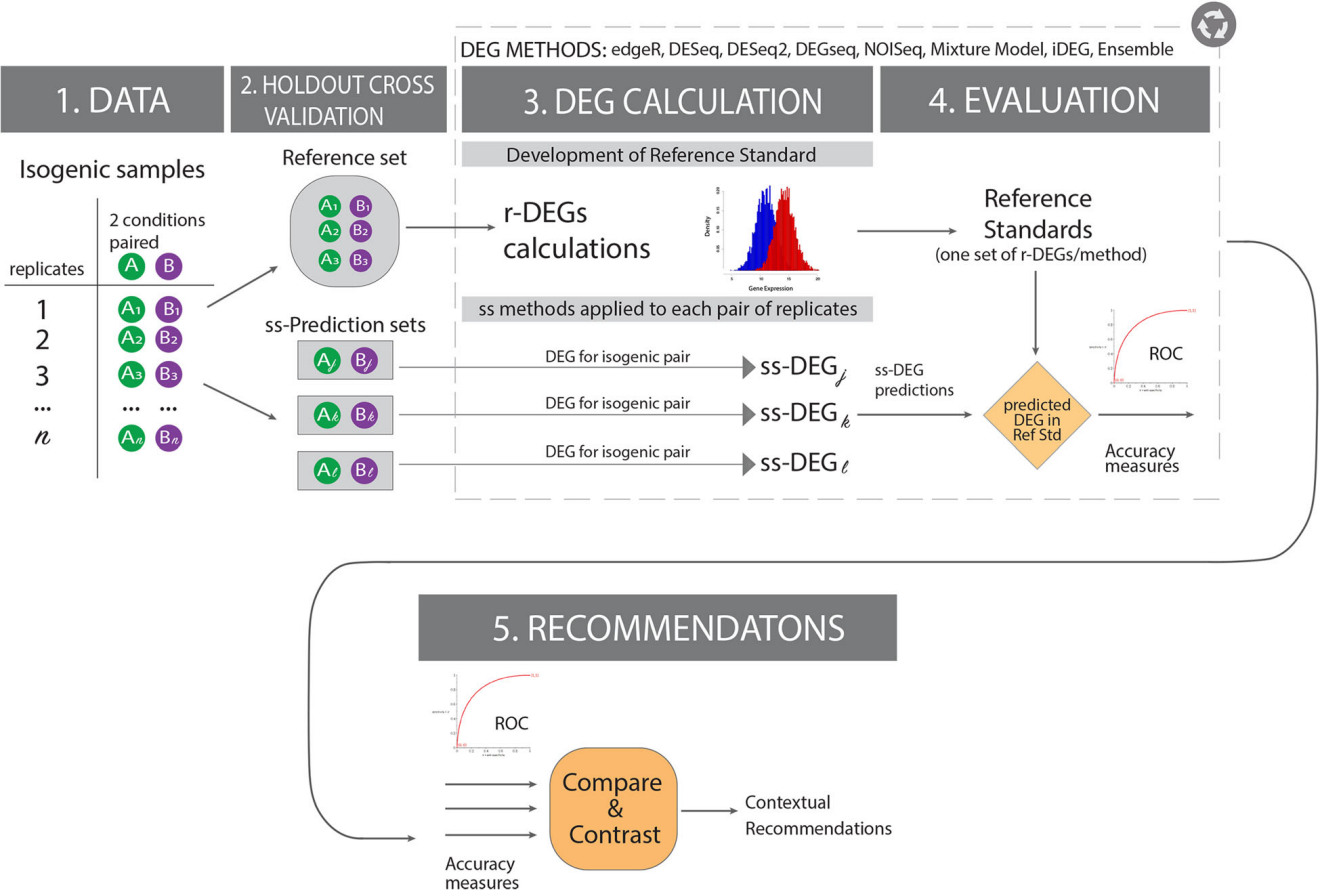
Evaluation strategy of methods designed for transcriptome analysis in paired single subject samples. *Motivation:* Identifying the gene products altered between two conditions in a single subject without replicates (ss-DEGs) is highly relevant in precision medicine. While conventional analytical methods may be applied to discover differences between isogenic *replicates* studied in distinct conditions (r-DEGs), precision medicine has helped usher in the possibility that diagnosis, prognosis, and therapeutic choices may be determined *more accurately from single-subject measurements*. Accurate ss-DEGs methods enable studying (i) cancer vs unaffected adjacent tissue or (ii) an ex-vivo cellular provocation assay operating on relevant tissue with or without therapy. *Evaluation framework.* Step 1. A dataset comprising multiple biological replicates of isogenic transcriptomes observed on samples taken in distinct biological conditions is identified. Step 2 The replicates are split into two groups of independent samples: a reference set and a single-subject (ss) prediction set. Step 3. Each r-DEG method (e.g., EdgeR, DESeq, etc.) is applied independently to the reference set to generate multiple reference standards, as each method has biases and none can be truly considered as a gold standard (Step 3, top panel). The reference set consists of biological replicates between two conditions of isogenic samples, and is thus a proxy for studying and mimicking the isogenic biologic variation of one subject (and each set of r-DEGs is an attempt at becoming a gold standard. In parallel, each ss-DEG method is applied to independent pairs of samples (one in each condition) taken from the prediction set, each as a proxy to a single subject (Step 3. Bottom panel). Step 4. Accuracy scores are determined for each ss-DEG method against each r-DEG-derived reference standard. Step 5. Summary statistics are conducted across all experiments to determine the best ss-DEGs according to the conditions of application

Figure 1 provides an overview of the experimental design, including the methods and recommendation for using an ensemble learner approach to develop robust reference standards in ss studies.

### Computing environment

All analyses in this study were conducted in the R programming language, using R 3.4.0 [17], and all the code is freely available at http://www.lussiergroup.org/publications/EnsembleBiomarker .

### Datasets

In this study, two distinct isogenic RNA-Seq datasets [18, 19] were used to calculate the reference standards and to conduct the single-subject studies. Both datasets have previously been used to evaluate methods that determine differentially expressed genes (DEGs) from RNA-Seq, using cohort or groups of biological replicates (r-DEGs methods) rather than for determining the accuracy of single-subject DEGs (ss-DEGs methods) as in the current study. Furthermore, for the sake of reproducibility, we conducted no additional preprocessing steps and used the final published datasets as provided by the experimenters via their portals [18, 19]. The preprocessing and normalization techniques used can be found in their original manuscripts [18, 19].

Yeast dataset: The first dataset (hereinafter) “Yeast” is comprised of 48 wild-type yeast replicates (*Saccharomyces cerevisiae* BY4741 strain, WT) compared to 48 replicates of a Δsnf2 mutant generated on the same background. RNA-Seq analysis and mapping includes 7126 measured genes [18]. We followed the author’s data preprocessing guidelines and conducted our studies using their suggested 42 WT and 44 Δsnf2 ‘clean’ replicates. Normalized and preprocessed data were downloaded as prepared by the original authors from their GitHub repository, under their “Preprocessed_data” directory. Forty-eight expression count files were downloaded for the two conditions, respectively, retaining the “clean” replicates for analysis.

MCF7 dataset: Our second dataset consists of 7 biological replicates of human MCF7 cells (~ 22,000 measured genes) which were either treated with 10 nM 17β-estradiol (E2) or cultured as unstimulated controls [19]. We used the 30 M read replicates available in the MCF7 dataset, which is available open source online under the Gene Expression Omnibus repository [20] (id = GSE51403). Normalized and preprocessed datasets were downloaded on January 21, 2018.

### Preprocessing and prediction set construction

The Yeast and MCF7 datasets were used entirely as obtained in their author-processed formats as described above, with no additional pre-processing steps or data manipulation. Transcript mapping, filtering, normalization, and batch correction details can be found in the original publications [18, 19]. In the MCF7 dataset, the following 4 biological replicates (“565–576”,“564–572”,“566–570”,“562–574”) were randomly selected as the reference set, with the remaining 3 (“563–577”,“568–575”,“569–571”) used to construct and evaluate how well the ss-DEG methods could recapture the reference-derived signal. Similarly, in the Yeast dataset, 30 replicates were randomly selected to construct the reference standard, with the remaining available 12 replicates used in the single-subject studies.

### DEG methods

**Fig. 2:**
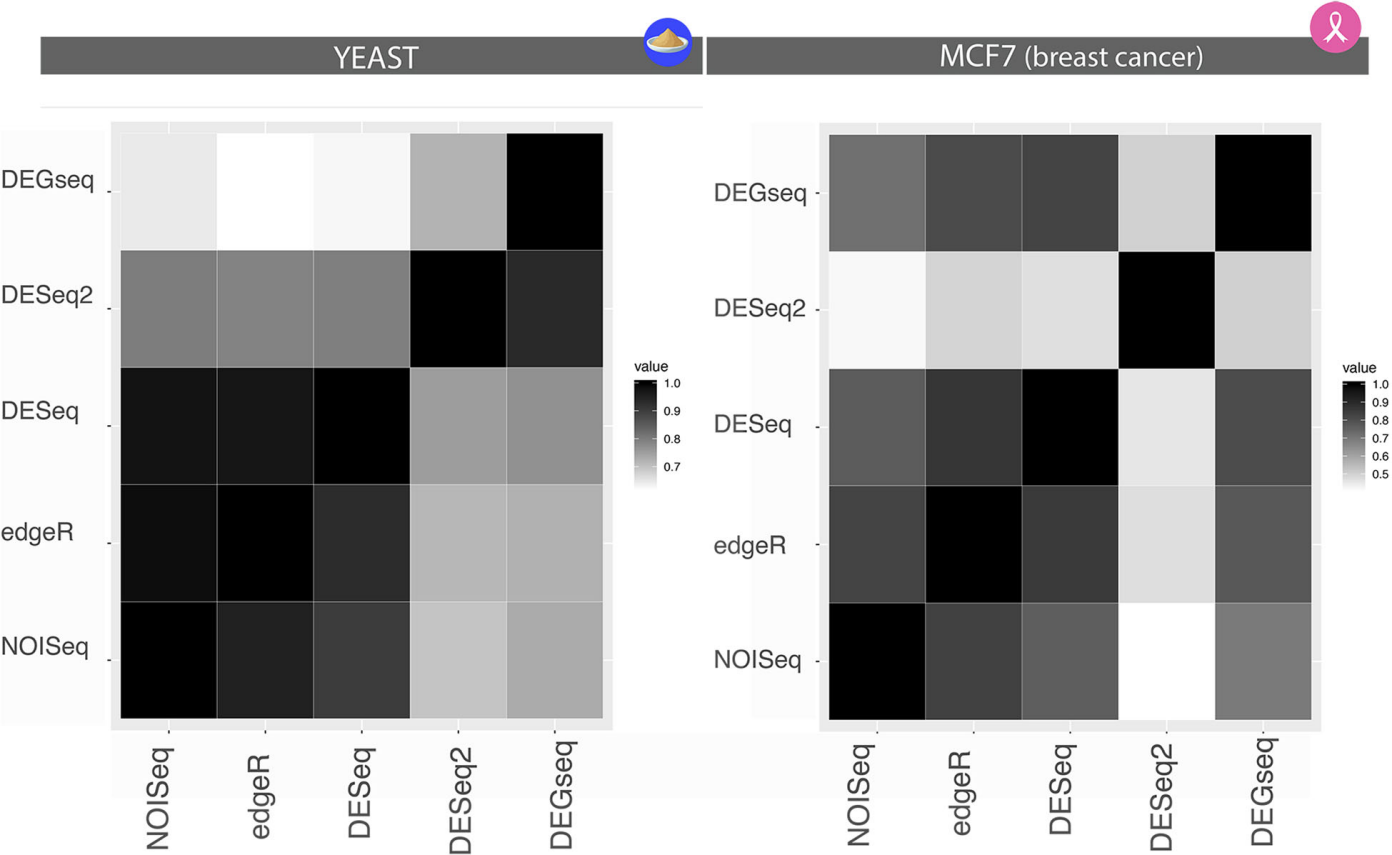
Reference Standards demonstrates high concordance between some techniques and major inconsistencies among others. Each method’s pairwise concordance with one another (identity overlap of DEGs) is shown, with the diagonal entries as the total number of DEGs of each respective method, demonstrating the vulnerability of studies relying on a single method to develop a reference standard. The pairwise intersections were calculated using the count of DEGs in the methods of each column as the denominator. The heatmap is approximately symmetric given the different denominators of comparing edgeR’s intersection with NOISeq vs. comparing NOISeq’s intersection with edgeR. In both Yeast (*n* = 30) and MCF7 (*n* = 4), edgeR, NOIseq, and DESeq show the best concordance to one another, while DESeq2 has the least concordance to any other method. DESeq2 shows the lack of agreement between what it considers DEGs and the rest of the methods, whereas in the left panel, both DESeq2 and DEGseq differentiate themselves from the cohort. This highlights the need for a consensus as some methods might make certain DEG calls that other methods miss and vice-versa. A conservative approach would be the intersection of all whereas an anti-conservative approach would take the union

The study is designed to better understand how single-subject studies can be conducted in biological and clinical precision medicine settings, where a true gold standard accurately reflective of a known ground truth does not always exist. To this end, we compared published and novel computational methods designed to detect DEGs from single-subject without replicates (ss) with a variety of well-validated and widely-used RNA-Seq analysis methods designed to identify DEGs from cohort or replicate-based comparisons (r-DEG) (Table 1) [5, 10, 15, 21–24]. With the exception of NOISeq that has been directly designed for application to a single subject under two conditions without replicates (NOISeq-sim implementation), the other replicate-based methods (Table 1) have not been designed nor systematically tested for accurate performance in single-subject, paired-sample conditions where replicates are not available. However, for the selected methods, the authors have estimated the required parameters to perform these comparisons, which are included in package documentation. All methods were implemented according to the default parameters provided for isogenic conditions (genotype-replicates) in the original publications. For NOISeq, we used noiseqbio function under their default parameter settings to generate the reference standard, and noiseq-sim (setting the parameters replicates = “no” and nss = 3) for the single-subject studies. For DESeq, in the *estimateDispersions* function, the method parameter is set to ‘per-condition’ for the replicated study, and ‘blind’ for the single-subject studies. For edgeR, we use the “genetically identical model organisms” replicate-type in order to set the appropriate BCV value; and finally, DEGseq and DESeq2 are implemented in wrapper functions using their default parameters. Figure 2 provides a graphical description of what methods were used to construct the reference standards for both datasets, illustrating the level of concordances between them.

**Table 1:** DEG Methods for Single-Subject Studies and their previous validations

Method	Experimental Design	Distribution Assumptions	*P*-value	Validation of method for single subject inference in original methods publications		
				*Internal*		*External*
				Simulation	Biological Replicates or Gold Standard	Translation to diagnosis, prognosis & treatment
edgeR [21]	r	NB	✓	✓^a^	✗	✗
DESeq [22]	r	NB	✓	✓^a^	✗	✗
DESeq2 [23]	r	NB	✓	✓^a^	✗	✗
DEGseq [24]	r	B	✓	✓^a^	✗	✗
NOISeq^a^ [15]	r/ss (as NOISeq-sim)	NP	✓	✓^a^	±^b^	✗
Mixture Model [5]	ss	MM	±^c^	✓	✗	✗
iDEG [14]	ss	NB	✗	✓^a^	✗	✗

*NB* Negative Binomial, *B* Binomial, *NP* Non-Parametric, *MM* Mixture Model, *ss* single-subject analytics, *r* analytics of between group of replicates, ✓ = completed, ± = partially addressed, ✗ = not addressed

^a^NOISeq-Bio was used to construct the reference standard, while NOISeq-sim was used in the single-subject prediction sets

^b^Partial validation conducted using qPCR with 400 genes with ~ 80% DEGs

^c^Mixture Model provides a posterior probability rather than a *p*-value, when FDR < 5% is indicated in the manuscript, it translates as a posterior probability > 95% for the mixture models

### ss-DEG calculations

**Fig. 3:**
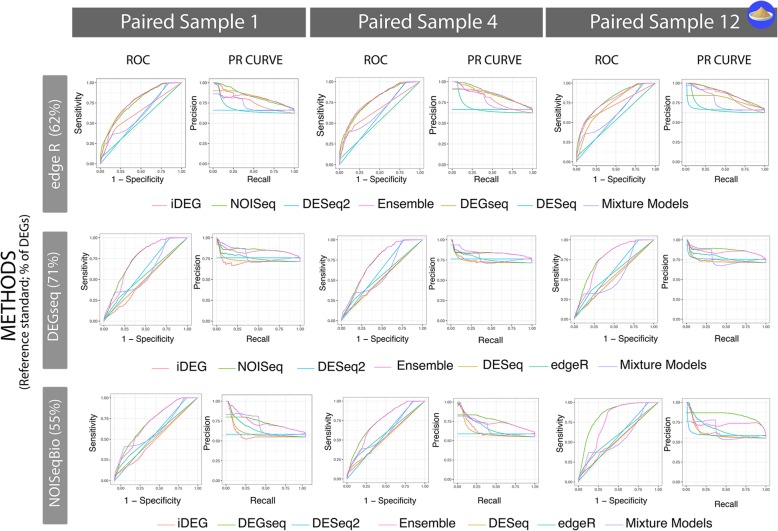
Exemplar accuracies of ss-DEGs methods validated using “All-against-One.” The selected seven ss-DEGs were evaluated against three rs-DEG-derived reference standards indicate a high-level of variability across ss-DEG methods and across reference standards, as well as a low to moderate-level of variability within ss-DEG methods and between biological replicates. The Precision-Recall and ROC curves across individual samples (Yeast) show that even in isogenic settings, a fair amount of biological variability exists. Furthermore, these single-subject studies provide a thorough comparison of each ss-DEG method’s performance and consistency in absence of replicates, allowing us to understand which tools have a greater potential for advancing precision medicine. For example, in the Yeast dataset, under > 40 biological replicates, the authors recommended DESeq and DESeq2. However, in absence of biological replicates, these techniques performed overly conservatively (unworkable recalls) and, on average, the worst

In this study, ss-DEG defines a class of methods, specifically each of the methods described in Table 1 when utilized in a single subject rather than applied across samples. For each DEG method in Table 1, we calculated ss-DEGs for 12 distinct pairs of samples in the Yeast prediction set, and 3 pairs of samples in the MCF7 prediction dataset. We did this by randomly generating pairs across conditions (i.e. selecting a random “WT” to pair with a random “snf-mutant” for Yeast, and “control” paired with “E2” for the MCF7 set) without replacement to ensure independence. Because each sample in the dataset is isogenic to all the others (save the presence/absence of the snf mutation characterizing the two conditions in Yeast), we can use this as a model for replicate pairs drawn from a single subject. As sample replicates drawn from the same individual, cell line, or model organism, they should follow identical distributions – with the exception of the DEGs results from the technical and biological errors and those attributable to the designed experimental differences. Of note, while many of the methods were not intended nor validated for ss-DEG calculations, the authors of each of the r-DEG methods (Table 1) did indicate their possible application to two-sample comparisons and provided unpublished approaches to adapt or estimate the parameters required for such processing. All details and code are available at http://www.lussiergroup.org/publications/EnsembleBiomarker. Figure 3 contains a set of exemplar precision-recall and ROC curves for the paired samples in the MCF7 dataset and the Yeast dataset.

**Fig. 4:**
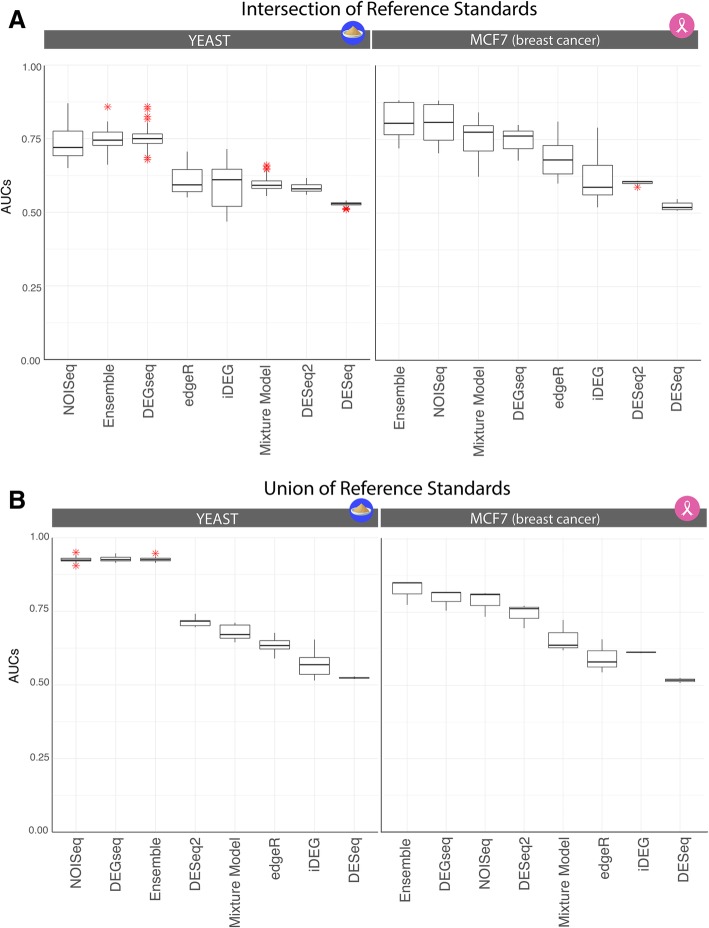
ROC summary plots in Yeast and MCF7. The Yeast case study produced reference standards that predicted between 55 and 70% of the genes in the genome as DEGs, while the MCF7 breast cancer cell lines predicted ~ 15% DEGs. In **a**, the intersection of all reference standard is used to produce what we would consider an “overtly-conservative” reference. The reference standard was constructed by taking intersection of the DEG lists from cohort analysis of the dataset with DESeq2, DEGSeq, edgeR, NOISeq-BIO (3118 genes as DE). Conversely, in **b**), the reference standard was constructed taking the union of all techniques (6425 genes as DE), resulting in an “anti-conservative” approach. The anti-conservative scenario facilitates the prediction task as a larger number of genes are called DEGs, which is advantageous to recall. In this case, methods like DEGseq stand out as they can maintain recall while not sacrificing precision since it will tend to call more genes as DEGs on average compared to its counterparts. DEGseq also operates invariantly at FDRs of 5–20%, making it highly suitable for precision medicine since an FDR of 5% is a default standard in clinical decision-making. In the overly conservative scenario with smaller number of DEGs in the gold standard, a more selective approach will perform better, highlighted in the precision parameter and illustrating the trade-offs available across all the tested techniques. An ensemble provides the analyst a robust trade-off alternative as it can build upon the strengths of all methods, and not suffer the issue of “performing well” in one dataset but not in another. In each panel, methods are ordered according to performance

**Fig. 5:**
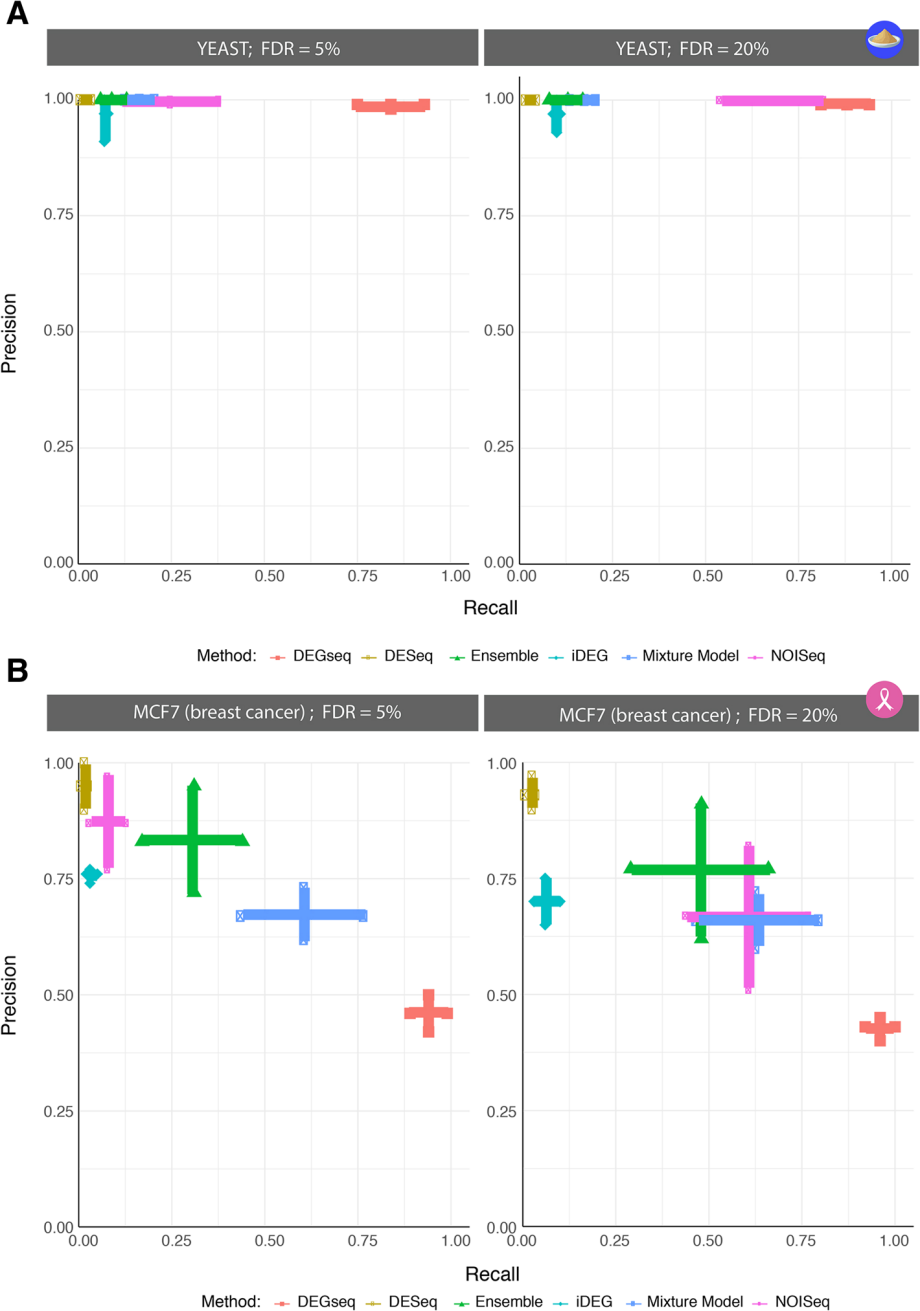
Precision-Recall summary plots in Yeast and MCF7 breast cancer cell lines. These aggregate results were constructed by summarizing precision-recall confidence regions over every ss-DEG evaluation by reporting the best mean values with one standard deviation bars in each direction creating a cross, to create the broadest possible precision-recall combinations. The curves show a spectrum of operating characteristics across techniques, indicating the need for an ensemble-like approach and substantial improvements in ss-DEG. The MCF7 case study produced reference standards that predicted between 15% of the genes in the genome as DEGs, while the Yeast case study produced reference standards that predicted between 55 and 70% of the genes as DEG. The more clinically relevant range of DEGs from the MCF7 reference standard construction introduces a very distinct detection problem where methods like DEGseq result in a large number of False Positive as shown in the precision-recall summary plots. It achieves high recall at the expense of low-precision. Conservative techniques like DESeq obtain a very high precision on a small number of calls. The results show this is a challenging detection task, and that various techniques operate differently, providing an analyst with a wide-range of operating characteristics. In the Yeast dataset, all methods achieve a high precision, with varying levels of recall, however given that the majority of genes are labeled DEGs, this favors methods with high number of calls. Since certain methods can perform well in one scenario and underperform in others, we recommend a contextual use or an ensemble-like approach where the strengths of these tools can be combined into a single, robust predictor. Here, precision and recall of each instance of ss-DEGs are respectively calculated on the union and the intersection of reference standards (Table 2). Of note, at FDR < 20%, DESeq2 produces no predictions and is thus not shown and considered inappropriate for single-subject DEG analyses.

False Discovery Rates (FDRs) were calculated using Benjamini-Yekutieli [25] given the dependent structure of the hypothesis tests. Mixture Models were implemented as described by Li et al. [5] and a posterior probability rather than a FDR is utilized for the receiver-operator characteristics curves and the precision-recall plots. In Figs. 4 and 5, the posterior probability > 95% of a fold change between two samples being a significant DEG was utilized as a Mixture Model cutoff corresponding to the FDR < 5%.

### Developing an ensemble learner across ss-DEG methods

Since differences across individual techniques showed variable performance, we constructed a naïve ensemble predictor (hereinafter referred to as the “ensemble”) which is an aggregate collection of multiple predictors. We adopted the same strategy of creating an ensemble out of multiple predictors from the popular and highly successful random forest algorithm [26] due to their high level of success in genomics. Continuing to treat each independent single subject as an independent assay, the ensemble combined ss-DEG predictions from DEGSeq, NOISeq, mixture models, and edgeR by taking the arithmetic mean of the FDR corrected values.

Formally, the ensemble prediction of DEG status of a gene *g*, noted *Ensemble (g)*, was constructed from multiple DEG methods *m*_*j*_ as:

1 $$ Ensemble\ (g)=\frac{1}{\left|M\right|-1\ }\sum \limits_{m_j\in M} fdr\left({m}_j\right)\times I\left({m}_j\right) $$

(where) $$ I\left({m}_j\right):=\left\{\begin{array}{c}1\ \mathrm{if}\ {m}_j\ne {m}_r\\ {}0\ \mathrm{if}\ {m}_j={m}_r\end{array}\right. $$

where *m*_*r*_ is the method used to build the reference standard, and *M* is the set of FDRs from “*m*_*j*_*”* models used to build the ensemble (i.e., *M* = {DEGseq, NOISeq, mixture models, edgeR}), *fdr*(*m*_*j*_) is the false discovery rate predcited by model *m*_*j*_ for a specific transcript *g*, |*M*| is the cardinality of *M* (e.g., the number of models), *I*(*m*_*j*_) is the indicator function of a subset of *M*. The reference standard is omitted from the construction of the ensemble in order to minimize any of its potential biases or unfair advantages since a reference set built from the specific algorithm “*m*_*x*_” will contain the same biases as a prediction set also constructed from the specific algorithm “*m*_*x*_”.

For example, when edgeR was used to build the replicated reference standard, the ensemble omits edgeR, and Eq. 1 becomes the case-specific Eq. 2:

2 $$ {\displaystyle \begin{array}{c} Ensemble\ (g)=\frac{1}{\left|M\right|-1\ }\sum \limits_{\begin{array}{c}{m}_j\in M\\ {}{m}_r= edgeR\end{array}} fdr\left({m}_j\right)\times I\left({m}_j\right)\\ {}=\frac{1}{3}\ \Big(\  fdr(edgeR)\times 0+ fdr(DEGseq)\times 1+ fdr\left( mixture\ models\right)\times 1+ fdr\left(\  NOIseq\right)\times 1\end{array}} $$

Since the single-subject implementations of both DESeq and DESeq2 had extremely low recall (recall< 1% of DEGs; Results, Figs. 3, 4, 5), these were excluded from the set *M* of candidate models. Finally, since iDEG [14] is currently a preprint publication, we decided against including it in the ensemble in order to create an ensemble consisting exclusively of published and peer-reviewed techniques.

### Reference standard construction

Each r-DEGs method in Table 1 was used to construct a reference DEG standard once using *n* = 30 wild type versus *n* = 30 snf2 mutant yeast for the Yeast dataset, and *n* = 4 unstimulated vs *n* = 4 estrogen-stimulated in the MCF7 dataset. DEGs identified by each r-DEG method were compared against one another to assess cross-method overlap for quantifying the variability and reliability of reference standards. All r-DEG methods were implemented using their recommended default settings as described earlier in the “DEG Methods” section.

In the original manuscript describing the MCF7 dataset [19], the authors set a threshold resulting in approximately 3300 genes detected as DEGs by edgeR when all 7 replicates were used. Therefore, we adjusted our False Discovery Rate (FDR) [26] thresholds in each method to operate similarly and detect approximately 3300 DEGs (~ 15% of genes). In the Yeast dataset, we mimicked the authors’ experimental design and set our FDR-thresholds for DEG detection at FDR < 5%, which resulted in a varying number of DEGs per method that closely resembled their results (e.g., number of DEG calls) obtained by the original authors analysis of this dataset. Table 2 summarizes the operating characteristics of these methods in both datasets.

**Table 2:** Generating Reference Standards with r-DEG methods in datasets with replicates Standards

Method	Yeast (*n* = 30 paired samples), genome size = 7126 genes			MCF7 (*n* = 4 paired samples), genome size = ~ 22,000 genes		
	FDR Threshold	Number of DEGs	Percent of Genome as DEG	FDR Threshold	Number of DEGs	Percent of Genome as DEG
edgeR	.05	4437	62%	.005	3231	14%
DESeq	.05	4594	64%	.001	3207	14%
DESeq2	.05	4802	67%	.0005	3255	15%
DEGseq	.05	5087	71%	3.56e-12	3351	15%
NOISeq	.05	3914	55%	0.078	3397	15%
Intersection of all methods	n/a	3118	44%	n/a	1173	5%
Union of all methods	n/a	6425	90%	n/a	6039	27%

FDRs are adjusted to obtain lists of DEGs of the same length as reported in the original publications. As shown with the intersection of all DEGs predicted by distinct methods, determining a gold standard in RNA-Seq analyses of multiple biological isogenic replicates remains a challenge

*n/a* not applicable

### “All-against-one” evaluation

In this study, we implemented an *all-against-one* evaluation framework as follows (Algorithm 1):

1. Choose one method in Table 2 and create the reference standard using the reference set and multiple replicates for each condition.
2. For all remaining other methods in Table 1, identify DEGs using a single pair of samples (one in each condition) from a separate, non-overlapping prediction set. Thus the methods for predictions are distinct from the one used for the reference standard.
3. All DEG predictions in step (2) (two conditions without replicates, i.e. two samples) were evaluated against all the unrelated reference standard built in step (1),
4. Repeat steps (1–3) for all methods in Table 2.

The “all-against-one” framework is conceptually akin to a leave-one-out (LOO) cross-validation [27] evaluation where instead of leaving out one sample, you leave out one method for identifying DEGs, and then evaluate it against the rest. This provides a more robust and honest evaluation in absence of a gold standard.

Algoritm 1 in detail, each of the replicated methods in Table 2 were used to construct a reference standard using a reference set. At each iteration, once the reference set was built, the remaining replicates were set aside as a prediction set. Then, each of the single-subject methods in Table 1 were evaluated in single-subject studies (ss-DEG) using the replicates as a prediction set (12 pairs of single-subject samples for Yeast and three for MCF7) using Precision-Recall (PR) and receiver-operator characteristic (ROC) plots. Finally, the method that was used to construct the reference standard was removed from the prediction set, to honestly report their accuracies against other techniques. Figure 3 illustrates the “all-against-one” evaluation. Note that when a method from Table 1 was used to construct a reference standard, the single-subject implementation of that same method was omitted from that series of analyses (i.e., in the reported summary statistics of accuracies of ss-DEGseq, the reference standard based on DEGseq was omitted from all precision-recall and accuracy metric evaluations).

Our predictions consist of three independent single-subject studies in the MCF7 dataset and 12 single-subject studies in Yeast that were not used for reference standard construction. Each was evaluated against five methods for replicate-derived reference standards, lead to 15 (MCF7) + 60 (Yeast) sets of PR and ROC curves (see Fig. 3 for an illustrative example). Each set of PR and ROC curves comprise 8 DEG methods generating predictions from two samples without replicates (550 PR and 550 ROC curves, because a method is not evaluated against its related reference standard, see Algorithm 1). Therefore, in order to meaningfully evaluate the methods across all conditions, we summarized each technique’s performance by analyzing their area under the curve (AUC), by calculating the AUCs in the PR and ROC curves. Furthermore, we illustrate each method’s operating characteristics by creating PR confidence regions which are 1-standard deviation (SD) bands around their mean precision and recall, at FDR = 5, 10, and 20% (1% also calculated, not shown).

### Summarizing results using union and intersection of gold standards

In Figs. 4 and 5, the *union* and *intersection* of reference standards (Table 2) were utilized to establish the summaries of accuracies of the “all-against-one” evaluation. Note, that the union and intersection are not necessarily biologically meaningful, since they may lead to overtly conservative or extremely anti-conservative DEG calls (e.g., the yeast data union produces 90% of the genes as DEGs). However, they do provide us with:

1. An illustrative example of best-case and worst-case scenarios (i.e., the extreme of possibilities).
2. A complementary illustration to Fig. 2 showing the lack of concordance across methods.

The PR and ROC plots were generated using the *precrec* R package [28] and the boxplots were created using the *ggplot2* [29] graphics library in R.

## Results

Evaluating DEGs between two conditions in a single subject without replicates has not been previously conducted using biologic samples. As previously reported by other authors, constructing a reliable reference standard from RNA-seq analytic methods remains a challenge [30] even in the presence of 30 replicates in each condition as in the Yeast dataset. As shown in Fig. 2, NOISeq, edgeR, and DESeq were the most concordant and robust methods for creating a reference standard. However, the overall concordance between all methods varies substantially (Table 2). For example, the authors of the original Yeast dataset report ~ 60% DEGs, while the union of all methods identifies as many as 90% DEGs, but their intersection reports a mere 44%.

Since no single reference standard is fully a statement of truth, nor their union or intersection, we systematically evaluated methods discovering DEGs in two conditions without replicates against all reference standards using the aforementioned “all-against-one” framework. As discussed in the Methods, distinct samples were utilized for calculating the reference standard and for estimating DEGs between paired transcriptomes. Figure 3 demonstrates nine out of the possible 420 PR and ROC curve combinations for the Yeast dataset (5 reference standards × 12 independent sets of two paired samples × 7 methods evaluations in estimating DEGs from two conditions without replicates). The 420 Yeast PR and ROC plots and the 105 MCF7 PR and ROC plots are respectively summarized in Figs. 4 and 5. In Fig. 4, the ROC curves are summarized using boxplots, and in Fig. 5, the PR curves are summarized into ‘average’ PR curves with a 1-SD band above/below and right/left of its mean precision-recall coordinate for both FDR 5 and 20%. As FDR increases, the techniques increase their recall at the expense of some precision, with the exception, of DEGseq whose precision and recall in the Yeast dataset minimally increases. DEG detection methods like Mixture Model and DEGseq perform fairly consistently across all samples, resulting in narrower confidence regions whereas NOISeq and iDEG’s variability lies on the higher end of the spectrum. Note, DESeq2 is not shown in Panel B neither in Fig. 4 nor in Fig. 5 given its failure to produce any predictions at the selected FDR cutoffs.

## Discussion

Our analyses clearly demonstrated the intricacies of working with biologically complex transcriptomic data in the absence of ground truth. As shown by Figs. 4 and 5, NOISeq-sim outperformed other tested single-subject techniques in terms of precision across both case studies and was capable of scoring well across a range of cohort-derived reference standards. In contrast, single-subject implementations of DESeq and DESeq2 were highly conservative. In addition, ss-DESeq2 does not perform without replicates, as in our hands, the method predicted zero DEGs when applied to either the Yeast or MCF7 single-subject sets, even though robust responses were noted by both other ss-DEG methods and cohort analyses suggesting a biological signal was present.

In the presence of a true gold standard, the kappa interrater agreement [31] could be utilized to compare methods, and precision and recall could be calculated more reliably without the requirement of creating method-specific reference standards. In absence of this, proper validation must be conducted to avoid misrepresenting the accuracy of the attained results. One major statistical issue with the way biological validations are currently conducted is that results typically only show each method evaluated against itself rather than against a true gold or reference standard. For example, in the MCF7 study, edgeR was determined to be the best technique using a reference standard built from edgeR, but not a reference standard built from a consensus. This evaluation better answers the question, “Which technique is best able to recapture the signal identified by their own model?” rather than addressing the biological question, “Which technique can best identify the signal in the data?” Because all these DEG models assume a variety of [count] parametric and non-parametric distributions, different models catch different signals, and it would be naïve to believe that any one model is superior to address all possible research questions and designs. Therefore, if there is not a clear consensus on which model best captures the biological signal, any evaluation framework must consider an *all-against-one* evaluation or an ensemble approach for a more honest and robust evaluation.

The proposed *all-against-one* experimental setting is akin to a leave-one-out (LOO) [27] cross-validation set up where instead of leaving out one sample, one method is left out for identifying DEGs, and in order to evaluate it against the remainder (i.e., create reference standards from DEGseq, edgeR, and NOISeq and recapture their DEG calls using DESeq, and then repeat for each individual DEG method). The evaluation graphs in Fig. 3 show a subset of these individual experiments where edgeR, DEGseq, and NOISeqBio are evaluated in a *all-against-one* approach, with *all* single-subject methods separately making DEG predictions *against* the signal identified by the *one* method used to construct the reference standard. Of note, we propose a conservative framework where a single-subject method is not evaluated against a reference standard built from its related method applied to replicates. In other words, both the data and the method used to build the reference standard are indepdent from the tested single subject method and its data substrate. Curiously, the authors that generated these reference standard datasets and produced evaluations of r-DEG methods, compared these method to a reference built from the same method, likely reporting inflated and biased accuracy rates attributable to their anticonservative evaluation framework. Perplexingly, these authors also reported that distinct r-DEG methods did not agree on the predicted DEGs but did not consider evaluating a method performance using another as a reference standard.

The *ensemble learner* approach follows the school of thought in machine learning that an individual strong classifier (say a decision tree or neural network) is less accurate than a classifier built from aggregating a collection of weaker classifiers since it may risk being unstable. One popular and effective way to build an ensemble is by way of bootstrapping and aggregating individual predictors [or bagging for short] [32]. In decision trees, for example, one carefully pruned decision tree [33] may be better than any sub-tree in a random forest classifier, but a random forest classifier as a whole (which is built on bootstrap and subsampling theory) will almost surely beat any individually-pruned decision tree as well as have less variability in its predictions. In our study, we translated the *ensemble learner* framework into the single-subject DEG study by aggregating predictions from individual ss-DEG methods (i.e., aggregating edgeR, DEGseq, mixture models, and NOISeq predictions) into a single-subject ensemble (ss-ensemble) method for identifying DEGs.

This proposed *ss-ensemble learner* approach consistently obtained high overall accuracies which suggests that a combination of parameter and distribution assumptions can overcome some of the limitations and biases inherent to any one model, further enabling a more accurate consensus standard (Fig. 4a). We note that one other method, NOISeq, performed nearly as well and could be used interchangeably for the sole use of predicting DEGs in single subjects. Thus, we recommend an ensemble approach over an individual predictor given that the ensemble offers the same precise predictive abilities, but with the added bonus of being robust to multiple distributional assumptions and their violations. Furthermore, the inherent diversity in the individual learners that enter the model (some are nonparametric while others are parametric techniques, and the parametric techniques assume a different set of distributions), enriches the final classifier [34] and provides a more accurate representation of the true biology, rather than one specific method’s statistical representation of it. Therefore, rather than focusing on the advantages and disadvantages of different distributional and parametric assumptions, we believe that all of their strengths can be leveraged if used and evaluated in a comprehensive and conservative framework, like the proposed “all-against-one”. Individual techniques always run the risk of being optimal in one dataset and suboptimal in another, as assumptions may be violated or appropriate on a dataset-to-dataset basis. However, an ensemble and holistic evaluation framework mitigates these risks; though, we are aware that further studies in this direction are required to fully demonstrate this added benefit. Future work will also extend our evaluation of the ensemble framework to include bootstrapping, by sampling isogenic pairs with replacement.

From this study, it also appears that all ss-DEG methods are sensitive to the percentage of DEGs present in the reference set. Given this, the degree of perturbation, or range in number of DEGs expected in a pair of samples, can guide the method selection. The Yeast dataset was utilized due to its large number of replicates for the construction of independent test and validation sets; however, the range of DEGs observed as a consequence of deleting a component of the transcriptional machinery is clearly higher than expected between most paired clinical samples. On the other hand, the MCF7 dataset was limited in term of samples but still provides some insight on DEG ranges of 15–30%. We had no datasets to evaluate conditions with DEGs< 15%. As simulations and synthetic data can investigate a range of accuracies against a true gold standard, they can be prone to other biases and limitations. Li et al. [14] have implemented a comprehensive simulation of ss-DEG methods across 8000 tests in a companion study using a range of DEG proportions from 5 to 40%, assuming distinct distributions (Poisson or Negative Binomial) and modeling a variable mean to variance relationship observed from real datasets as recommended by McCarthy et al. [35]. The results from those simulations broadly agree with the results obtained in this study, identifying the same precision and recall rankings between NOISeq-sim, edgeR, DESeq, and DEGSeq when used with replicates to construct the reference standard. In contrast, however, simulation studies generally yielded higher recall estimates, suggesting that the observed residual cross-replicate heterogeneity comprised of non-genomic and stochastic variation of real biologic datasets can substantially limit performance of the DEG methods applied to two conditions without replicates. Due to this, we suggest that these methods’ performance should be viewed as a range or spectrum, rather than definite.

The union of the reference standards provides 90% DEGs, suggesting that our framework illustrates how anticonservative the accuracy rates reported in studies [18, 19] are, as each method was evaluated against itself in these previous studies. While 90% of DEG is biologically unrealistic, it wholly illustrates the extent to which all DEG methods disagree. Conversely, we also provide a conservative reference standard (intersection of methods). This again can produce extremely low percentage of DEG calls and is sensitive to the choice of algorithms used. These extremes show the need for a more robust and consistent framework for reference standards akin to that of an ensemble approach or the “all-against-one”; it also provides a lower and upper bound of DEG calls that can be expected in any biological study in order to best study the characteristics of the methods and data being analyzed. We propose using the “all-against-one” framework for future studies and the use of an ensemble to mitigate these challenges.

Based on the results shown in Figs. 4 and 5, we report in Table 3 recommendations for the use of ss-DEGs in two conditions without replicates. Of note, when comparing our results to the performance metrics published alongside the Yeast and MCF7 data in the original publications by Schurch [18] and Liu [19], we found that the performance was lower across our studies. This may be due to those authors calculating the accuracy of their r-DEG methods in the presence of replicates using anti-conservative conditions: each method was compared to itself using the total number of replicates, while substudies utilized a random sample within those utilized for the reference standard. Here, our accuracies are more conservatively calculated in two ways: (i) we constructed each reference standard by using distinct samples for predictions without predicates from the reference standard construction, and (ii) the accuracy scores of a method predicting DEGs without replicates were tested against reference standards built by distinct methods in replicates.

**Table 3:** ss-DEG Methods Recommendations: Single-subject studies of two-sample conditions without replicates

	Combinations of accuracies															
	15% < DEGs < 30%												55% < DEGs < 70%			
Precision (%)→	> 90%				> 70%				50				> 90%			
Recall→ (>%)	90	70	50	25	90	70	50	25	90	70	50	25	90	70	50	25
Methods																
Ensemble	✗	✗	✗	✓	✗	✗	✓	✓	✗	✗	✓	✓	✗	✗	✗	✓
NOISeq	✗	✗	✗	✓	✗	✗	✓	✓	✗	✗	✓	✓	✓	✓	✓	✓
DEGseq	✗	✗	✗	✗	✗	✗	✗	✗	✓	✓	✓	✓	✓	✓	✓	✓
Mixture Model	✗	✗	✗	✗	✗	✗	✓	✓	✗	✗	✓	✓	✗	✗	✗	✓
edgeR	✗	✗	✗	✗	✗	✗	✓	✓	✗	✗	✓	✓	✗	✗	✗	✓
iDEG	✗	✗	✗	✓	✗	✗	✗	✓	✗	✗	✗	✓	✗	✗	✗	✓
DESeq	✗	✗	✗	✓	✗	✗	✗	✓	✗	✗	✗	✓	✗	✗	✗	✓
DESeq2	✗	✗	✗	✗	✗	✗	✗	✗	✗	✗	✗	✗	✗	✗	✗	✗

✓ = recommended; ✗ = not recommended

Figure 4a shows how DEGseq performs similarly to NOISeq and the ensemble method maintains precision at FDR 5% and properly detects nearly 75% of the DEGs. One could argue this combination would potentially make DEGseq the ideal tool for this dataset; however, in the MCF7 case study, DEGseq could not replicate its performance (Fig. 5b). This highlights the risks of relying on a single technique across distinct DEG proportions. Furthermore, under multiple biological replicates, techniques like DESeq, DESeq2, and edgeR are the staples of RNA-Seq data analysis and are often the authors’ default method choices and recommendations for building reference standards. However, as seen in Figs. 4 and 5, DESeq and DESeq2 performed overly conservatively in the two datasets (extremely low recalls), showing that their reliability does extend to single-subject (without replicate) conditions. This study provides a promising first comparison of how RNA-Seq analysis techniques fare in comparing two conditions in absence of replicates (one sample per condition). In addition, ranges of DEGs< 15% - that were not explored here - also merit to be explored as they are likely clinically relevant in response to therapy. The addition of more datasets with additional response ranges would further improve our understanding of the accuracy of ss-DEG methods, especially when these datasets are previously validated, as was the case of the MCF7 and Yeast datasets. Furthermore, improvement of ss-DEGs methods is required, particularly for performing with higher recall when DEGs are low. Further studies are also needed to describe the effectiveness of better performing methods, such as NOISeq (specifically NOISeq-sim), in the absence of replicates across different contexts.

In the past, comparing a transcriptome to heterogenic samples from other subjects has been proposed. However, this strategy brings up a number of confounding factors: distinct genetics, distinct environmental factors, etc. Here, we proposed using one’s own samples as controls. Adding biological replicates increases accuracy and is recommended where possible, but may not be feasible in certain clinical settings and can be cost prohibitive. In the absence of replicates, focusing on identifying those DEGs within differentially expressed pathways may further improve the accuracy rates and also merits validation in future studies.

## Conclusions

This study demonstrates that determining differentially expressed genes (DEGs) between two conditions of one subject in absence of replicate samples (two samples total) can be obtained with high precision and limited recall (< 30%) when the true number of DEGs ranges from 15 to 30%, while a few methods can also provide reliable results under conditions where the proportion of DEGs exceed 50% of the genome. No single-subject ss-DEG method obtained both high precision and recall in the evaluations using these biological datasets, though some obtained a reasonably robust F1-score.

As RNA-Seq technologies expand the opportunities to analyze single-subject data, more time and research need to focus on a greater understanding of which analysis tools are better suited for clinical samples and individual inferences. At the moment, the limited access to a sufficient quantity of clinically relevant tissues typically prohibits replicate sampling. Thus, conventional analystical methods that require replicates to determine DEGs must be adapted or replaced in order to advance the utility of transcriptome profiling in precision medicine. This study demonstrates that ongoing improvements in single-subject methods are required for these to work robustly and accurately in absence of replicates. We have also shown that the biological and data characteristics of RNA-seq are also critical factors that affects method performance, as the relative strengths and limitations of each method differed markedly depending on the proportion of DEGs regulated by the bioassay. However, ensemble methods for single-subject analyses enabled consistent performance regardless of the studied conditions.

Further, it still remains difficult to generate consensus reference standards from different RNA-seq analysis tools as the intersection of all well-established methods agreed on less than 50% of called DEGs, even when implementing these tools under their recommended conditions with replicate samples in well-studied datasets. Previous studies [36] have shown the translation value obtained from using single sample data for clinical phenotyping, thus we must continue expanding the methodology and framework along this direction. In order to improve the accuracy, we propose that future methods consider the injection of knowledge from curated gene set (e.g., Gene Ontology) and network science (e.g., unbiased gene set obtained from co-expression networks) to pool the signal of altered genes belonging to functional units as a way to increase signal accuracy and reliability in single subjects While the reductionism of identifying directly DEGs from stwo samples is appealing, previous systems genomics work, showing stronger signals at differentially expressed pathways in single-subject studies, suggests combining the two approaches would substantially increase DEG accuracies.

## Abbreviations

AUC: Area under the curve
DEG: Differentially expressed genes
FDR: False discovery rate (specifically, BY)
PR: Precision-recall
r-DEG: Methods based to determine DEGs between two conditions using replicates in each condition
ROC: Receiver-operator characteristics
SD: Standard deviation
ss: Single-Subject
ss-DEG: Methods based to determine DEGs between two conditions without replicates in each condition (two samples total)
WT: Wild type
